# Supplementary material for: Kenny mediates selective autophagic degradation of the IKK complex to control innate immune responses
Source: Nat Commun. 2017 Nov 2;8:1264. doi: 10.1038/s41467-017-01287-9 (PMC5668318; doi:10.1038/s41467-017-01287-9)
Supplement: Supplementary file 1 — Supplementary Information [file 41467_2017_1287_MOESM1_ESM.pdf]

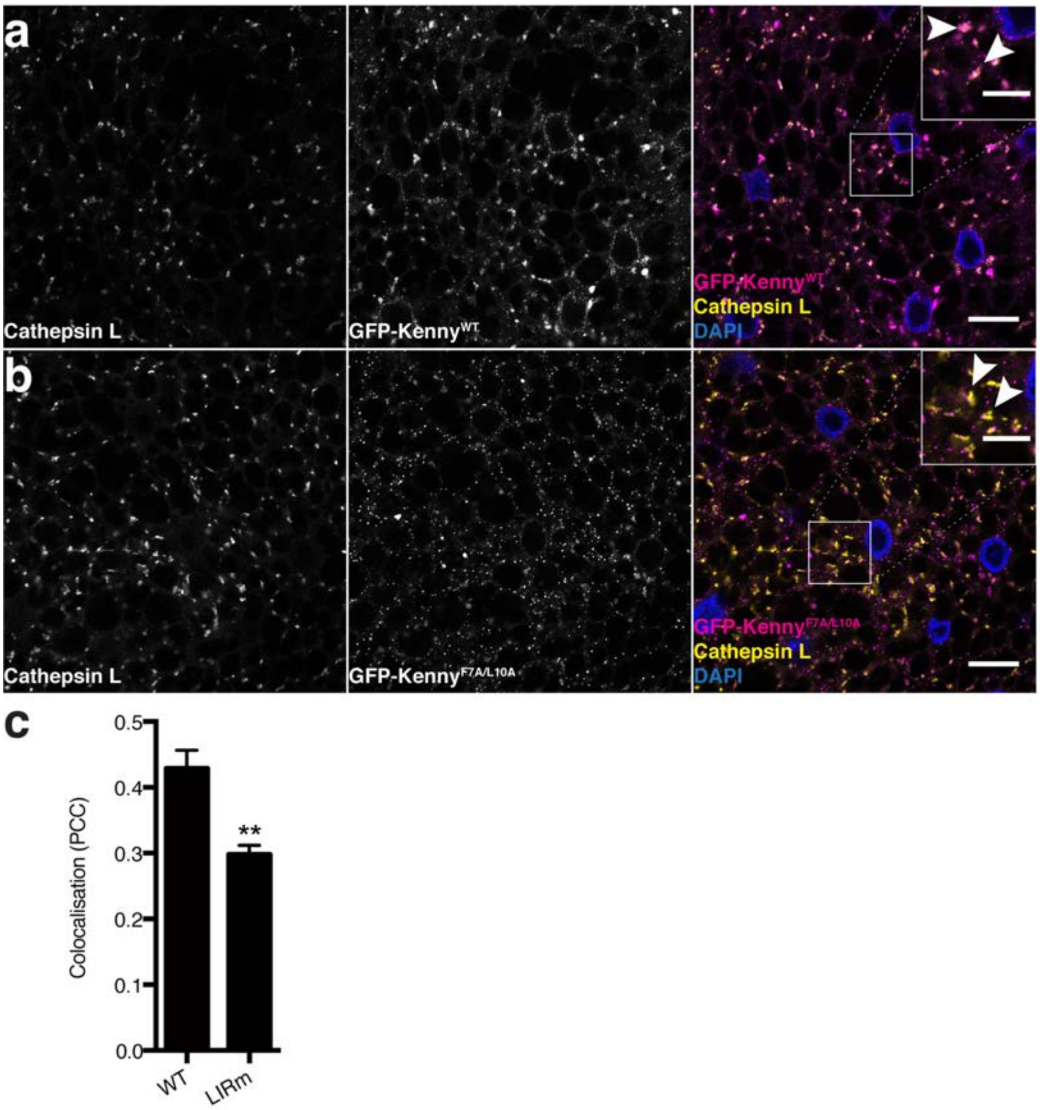

Supplementary Figure 1. The LIR motif in Kenny is necessary for its targeting to the lysosomes.

(a-b) Confocal sections from fat body cells from larvae expressing GFP-Kenny^{WT} (a-a”) or GFP-Kenny$^{F7A/L10A}$ (magenta) (b-b”). Larvae were starved for 4 hours to induce autophagy. Anti-Cathepsin L antibody was used to stain active lysosomes (yellow). Arrowheads show area of interest displaying either colocalisation between both protein (a) or lack of colocalisation (b). Scale bars are 20µm (original panels) and 10µm (insets). **(c)** Quantification of the colocalization of Cathepsin L and GFP-Kenny signals using the Pearson’s correlation coefficient (**P<0.01, two-tailed Student’s *t*-test; error bars, SEM).

Genotypes: (**a**) *Cg-GAL4/+;UAS-GFP-Kenny-WT/+*; (**b**) *Cg-GAL4/UAS-GFP-Kenny-F7A/L10A*.

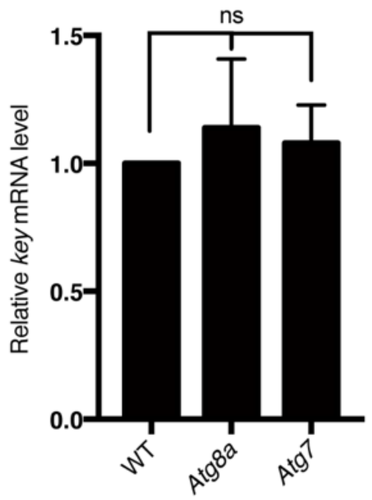

Supplementary Figure 2. Kenny (*key)* expression level is not affected in autophagy-deficient flies.

Analysis of *key* mRNA fold increase by RT-qPCR on RNA extracted from young adult flies. Bar charts denote mean ± s.d. (ns P>0.05, two-tailed Student's *t*-test).

Genotypes: w^{1118}, *Atg8a*, $Atg7^{\Delta 14}/Atg7^{\Delta 77}$

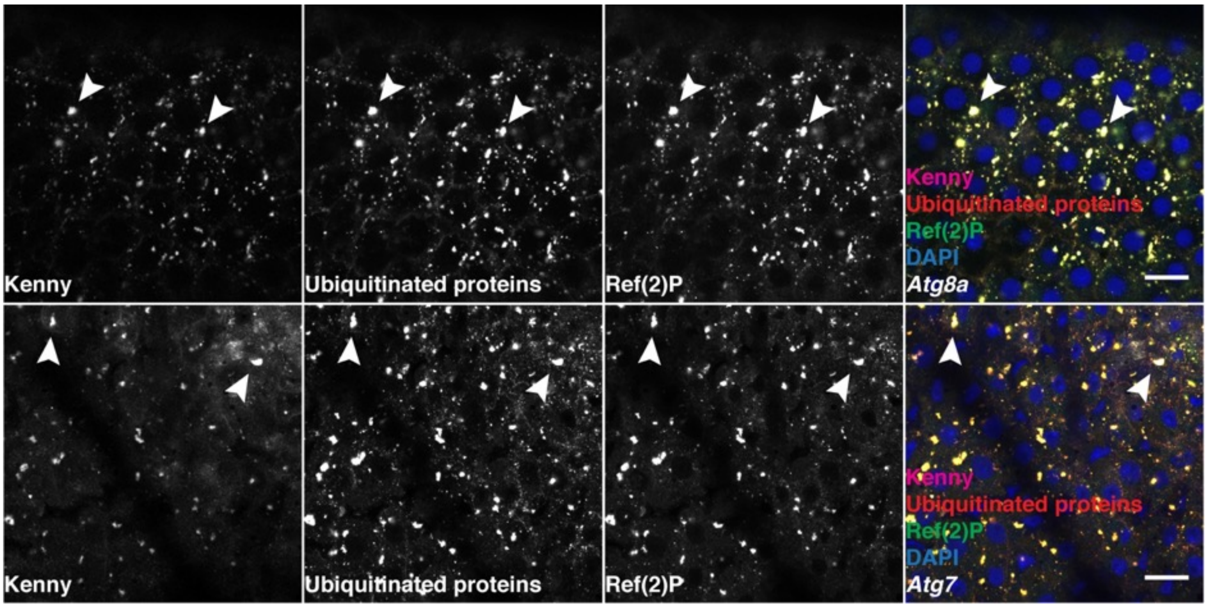

Supplementary Figure 3. Kenny colocalizes with aggregate markers in autophagy-deficient flies.

Confocal sections from midguts from adult *Atg8a*^{KG} (top panels) or *Atg7*$^{\Delta 14/\Delta 77}$ (bottom panels) mutants stained for Kenny (purple), ubiquitinated proteins (red), Ref(2)P (green) and nuclei (blue). Arrowheads show Kenny aggregates that colocalise with protein aggregate marker Ref(2)P and ubiquitinated proteins. Scale bars are 20µm.

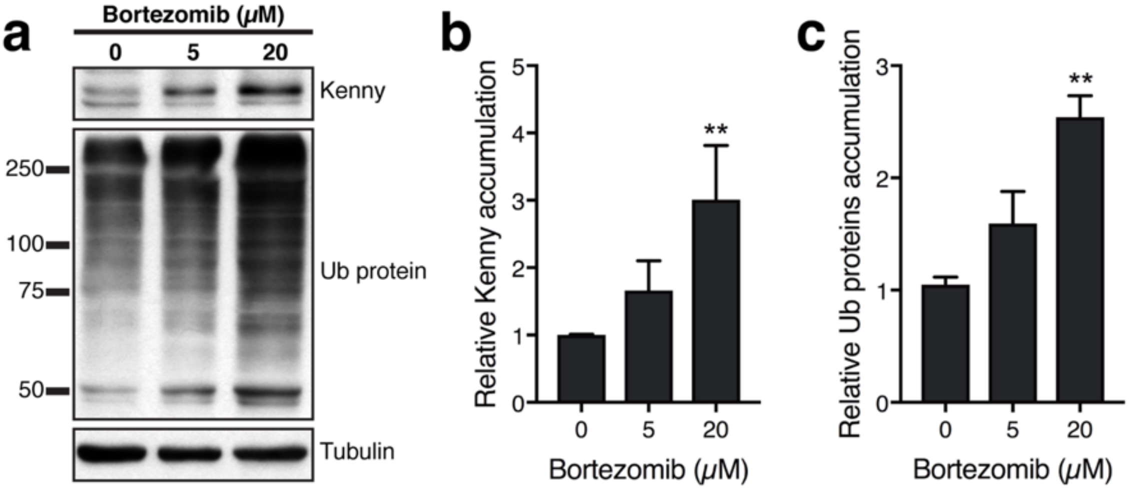

Supplementary Figure 4. Kenny is moderately degraded by the proteasome.

(a) Lysates from wild-type adult flies fed for 6 days on food supplemented with Bortezomib were subjected to SDS-PAGE and immunoblotting for Kenny and ubiquitinated proteins. Tubulin was used as a loading control. **(b-c)** Quantification of the accumulation of Kenny (b) and ubiquitinated proteins (c) normalised to tubulin. Bar charts denote mean ± s.d. (**P<0.01, one-way ANOVA).

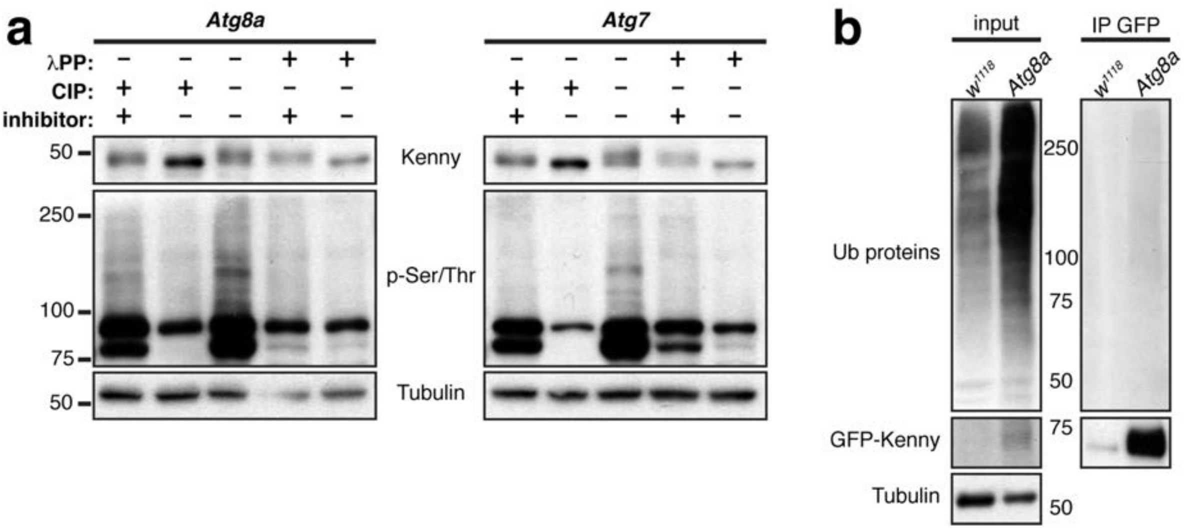

Supplementary Figure 5. Kenny is phosphorylated in autophagy mutant flies.

(a) Lysates from *Atg8a*^{KG} or *Atg7*$^{\Delta 14/\Delta 77}$ were treated with phosphatases (λPP or CIP) with or without addition of phosphatase inhibitor cocktail. Samples were subjected to SDS-PAGE followed by immunoblotting for Kenny and phosphor-Ser/Thr. Tubulin was used as a loading control. **(b)** Denaturated protein lysates from males were used for the immunoprecipitation of GFP-Kenny. Inputs and IP eluates were subjected to SDS-PAGE and immunoblotting for ubiquitinated proteins and GFP. Tubulin was used as a loading control for the inputs.

Genotypes: *w*^{1118}*/Y;tub-GAL4 UAS-GFP-Kenny/+*; *Atg8a*^{KG}*/Y;tub-GAL4 UAS-GFP-Kenny/+*

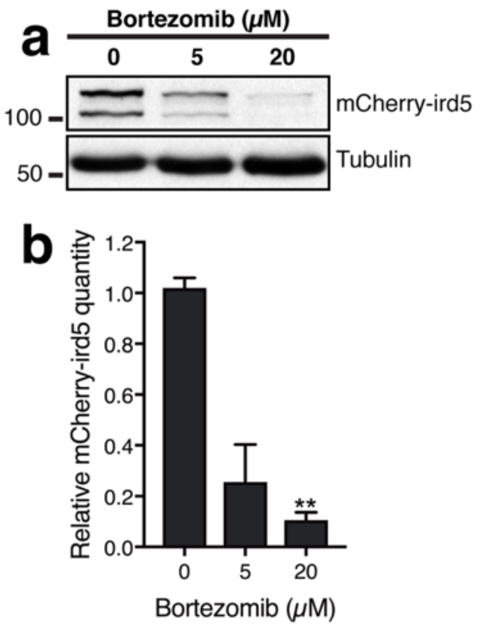

Supplementary Figure 6. mCherry-ird5 is not degraded by the proteasome.

(a) Lysates from adult flies fed for 6 days on food supplemented with Bortezomib were subjected to SDS-PAGE and immunoblotting for mCherry-ird5. Tubulin was used as a loading control. **(b-c)** Quantification of the protein levels of mCherry-ird5 normalised to tubulin. Bar charts denote mean ± s.d. (**P<0.01, one-way ANOVA).

Genotype: *Cg-GAL4 UAS-mCherry-ird5*

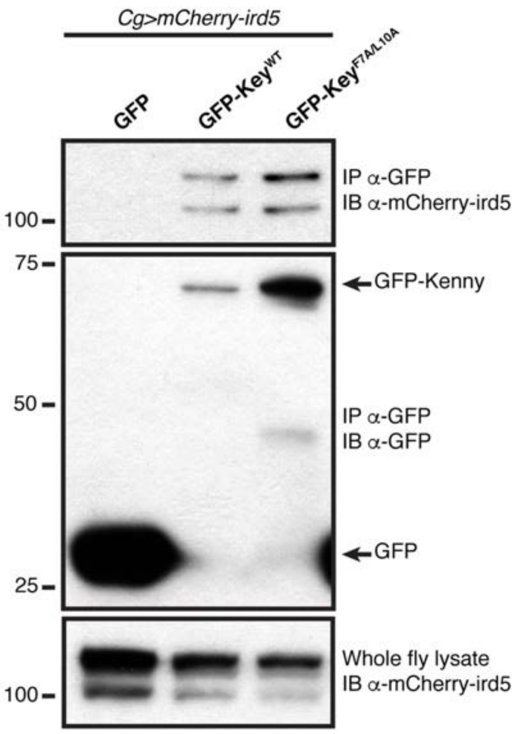

Supplementary Figure 7. Ird5 interacts with Kenny.

In vivo immunoprecipitation between mCherry-ird5 and GFP-Kenny-WT or –F7A/L10A. GFP or GFP fusion constructs of Kenny were immunoprecipitated (*IP*) from fly lysates and subjected to SDS-PAGE. *IB*: immunoblot.

Genotypes: *Cg-GAL4 UAS-mCherry-ird5/+;UAS-GFP/+*; *Cg-GAL4 UAS-mCherry-ird5/+;UAS-GFP-Kenny*^{WT}*/+*; *Cg-GAL4 UAS-mCherry-ird5/UAS-GFP-Kenny*$^{F7A/L10A}$.

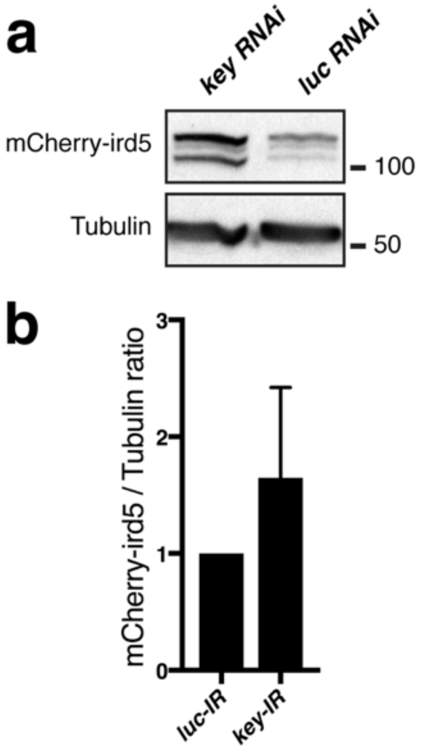

Supplementary Figure 8. mCherry-ird5 protein levels in *key* silenced flies.

(a) Lysates from flies expressing mCherry-ird5 along with RNAi against *luciferase* (control) or *key* were subjected to SDS-PAGE and immunoblotting for mCherry-ird5. Tubulin was used as loading control. **(b)** Quantification of mCherry-ird5 protein levels normalized to tubulin. Bar chart denotes mean ± s.d.

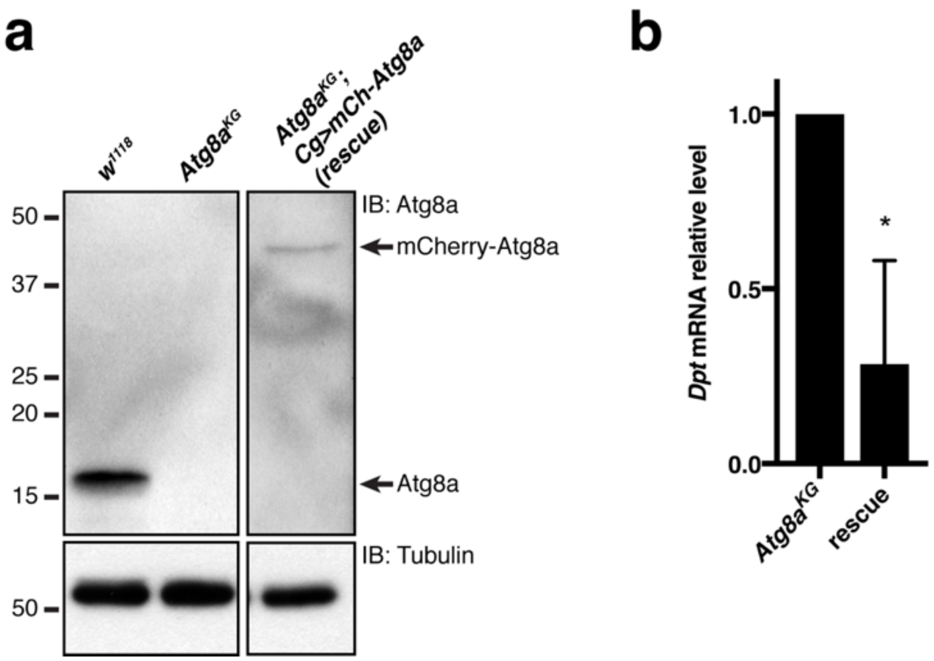

Supplementary Figure 9. Partial rescue of IMD pathway deregulation

(a) *Diptericin* upregulation observed in Atg8a mutant flies was rescued by expressing selectively mCherry-Atg8a in the fat body. Expression of the transgene was verified by western blotting against Atg8a protein. **(b)** Quantification by RT-qPCR of the relative expression of *Dpt* in Atg8a mutant and rescue adult flies. Bar charts denote mean ± s.d. (*P<0.05, two-tailed Student's *t*-test).

Genotypes: w^{1118}/Y, $Atg8a^{KG}/Y$ and $Atg8a^{KG}/Y;Cg\text{-}GAL4\ UAS\text{-}mCherry\text{-}Atg8a/+$

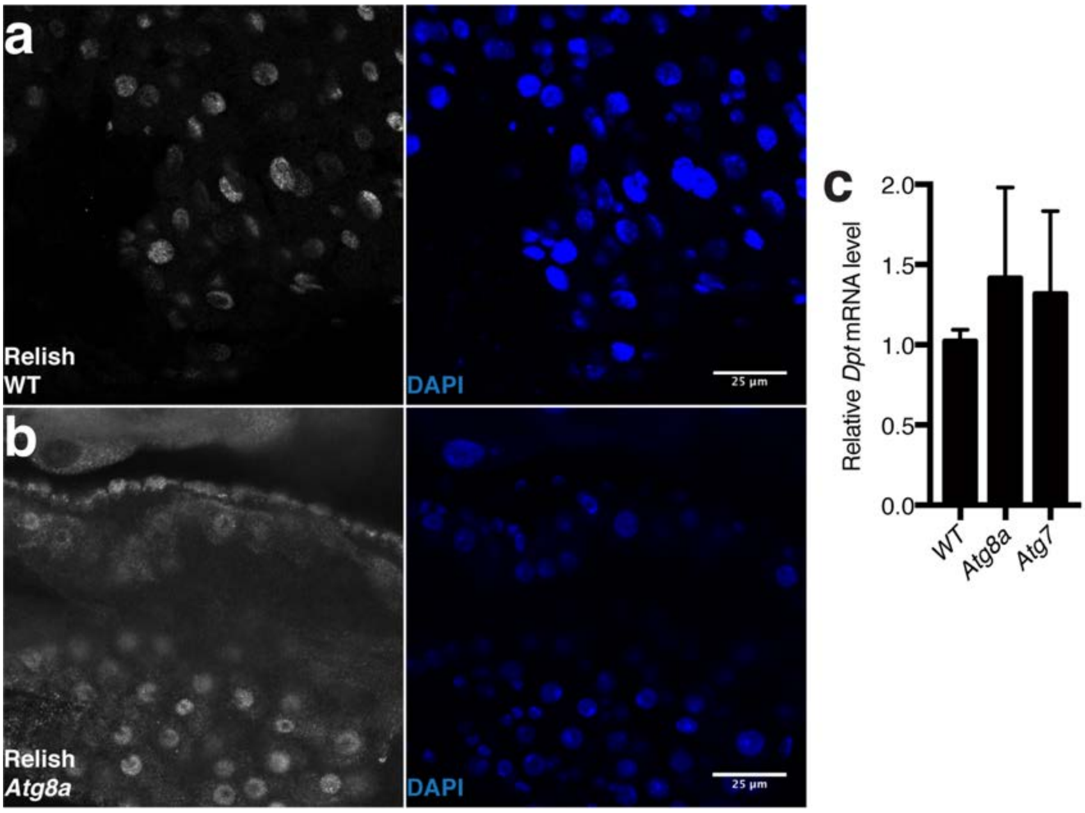

Supplementary Figure 10. Relish translocation and *Dpt* expression level are not affected in guts from autophagy-deficient flies.

(a, b) Confocal section of posterior midgut from adult wild-type and Atg8a mutant flies stained for Relish (grey) and nuclei (Hoechst, blue). Scale bars are 25µm. (c) Analysis of *Dpt* mRNA fold increase by RT-qPCR on RNA extracted from isolated young adult fly guts. Bar charts denote mean ± s.d.

Genotypes: w^{1118}, $Atg8a^{KG}$, $Atg7^{\Delta 14}/Atg7^{\Delta 77}$

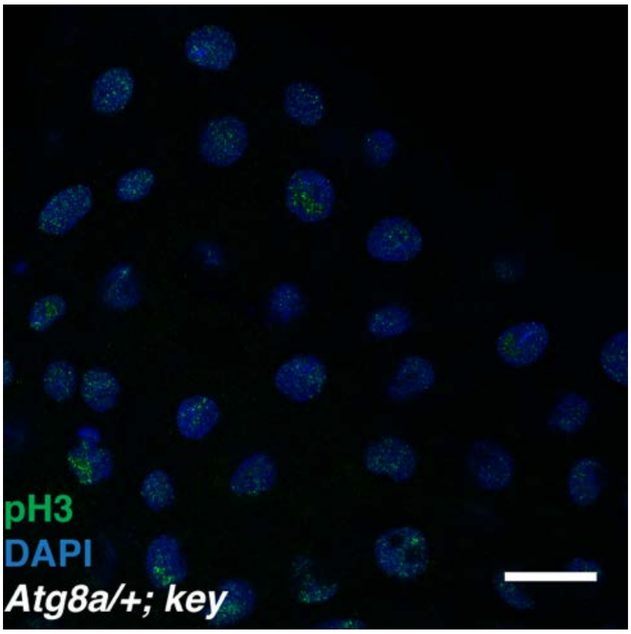

Supplementary Figure 11. No intestinal dysplasia in *Atg8a;key* mutant flies

Confocal sections of posterior midgut from double mutant *Atg8a;key* stained for phospho-H3 (pH3, green) and nuclei (DAPI, blue). Scale bar is 20µm.

Genotype: *Atg8a/+;key*^{1}

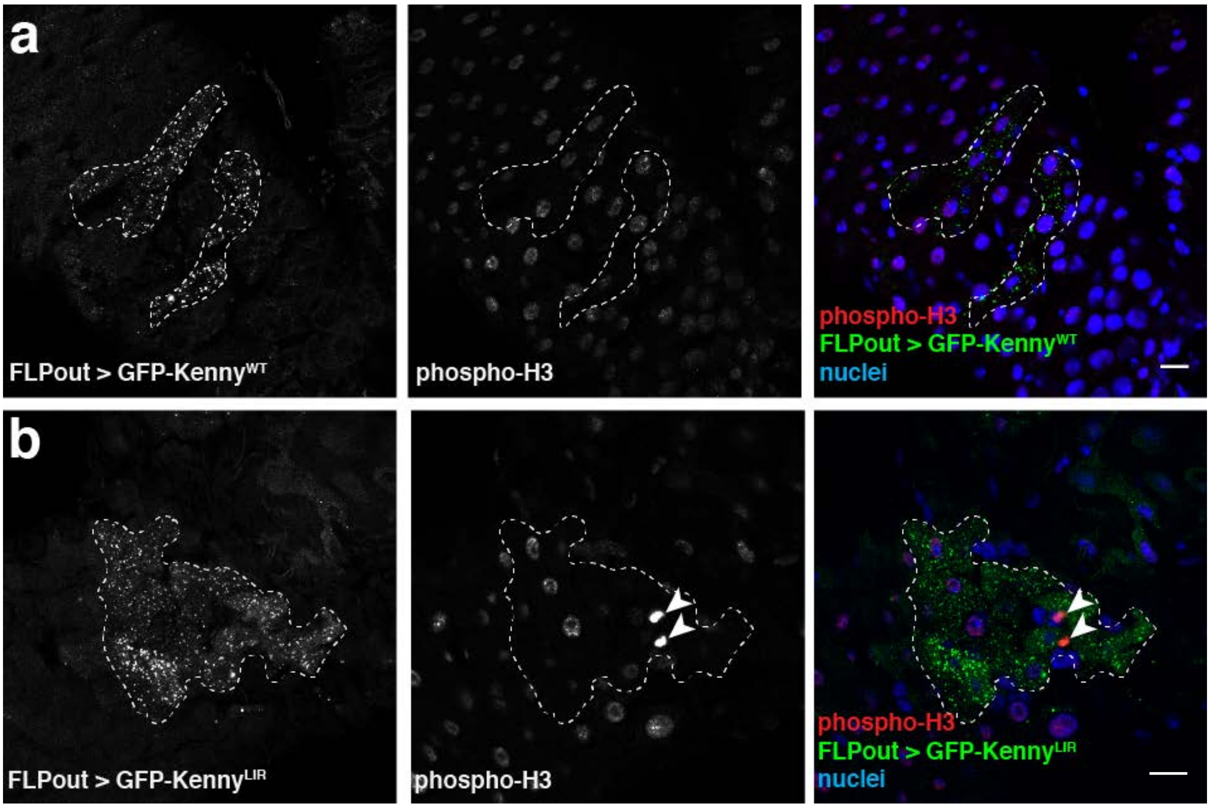

Supplementary Figure 12. Phospho-Histone 3 immunostaining in cells ectopically expressing LIR mutated GFP-Kenny. Confocal sections from adult posterior midgut clonally expressing GFP-Kenny wild-type **(a)** or LIR mutated **(b)** (green) and stained for phospho-H3 (red) and nuclei with Hoechst (blue). Arrowheads show pH3 positive cells in clones expressing LIR mutated GFP-Kenny. Scale bars is 10µm.

Genotypes: (a) *yw hs-flp/+;;UAS-GFP-Kenny*^{WT}*/Ac>CD2>GAL4*, (b) *yw hs-flp/+;UAS-GFP-Kenny*$^{F7A/L10A}$*/+;Ac>CD2>GAL4/+*

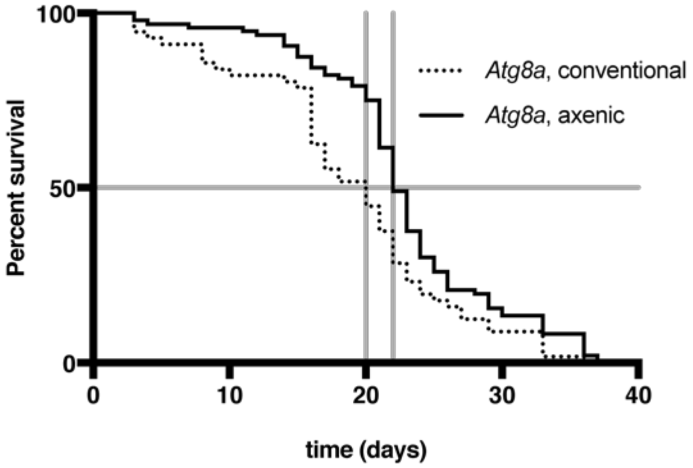

Supplementary Figure 13. Germ-free *Atg8a* mutant flies have a longer median lifespan than their conventionally-reared siblings.

The lifespan of a hundred *Atg8a* mutant males reared in conventional or axenic condition were monitored. P=0.0058, Log-rank Mantel-Cox test.

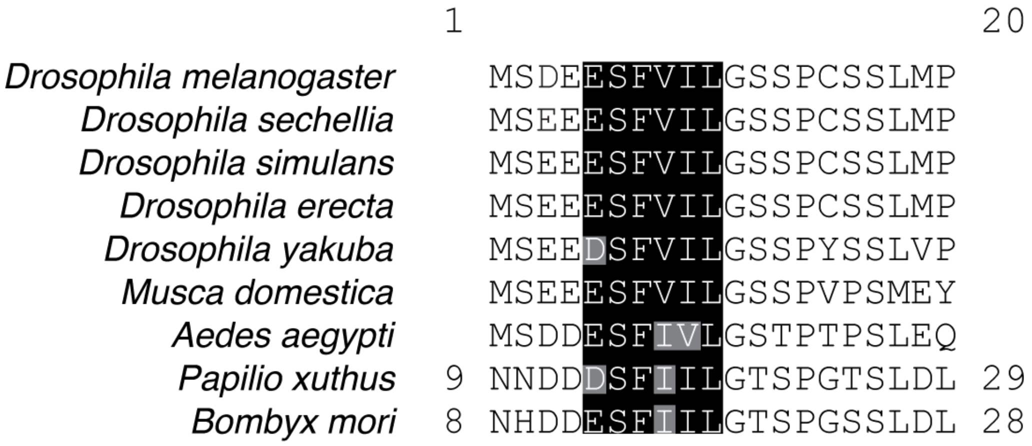

Supplementary Figure 14. Sequence alignment of the LIR motifs from various Kenny orthologues. FASTA sequence of Kenny was analysed through protein-BLAST in order to find orthologues in other species. Twenty amino acids from each sequence were aligned based on the position of the LIR motif, which is shown in black. Gray boxes indicate LIR residues which are different, but similar in size/charge to the ones in *D. melanogaster*.

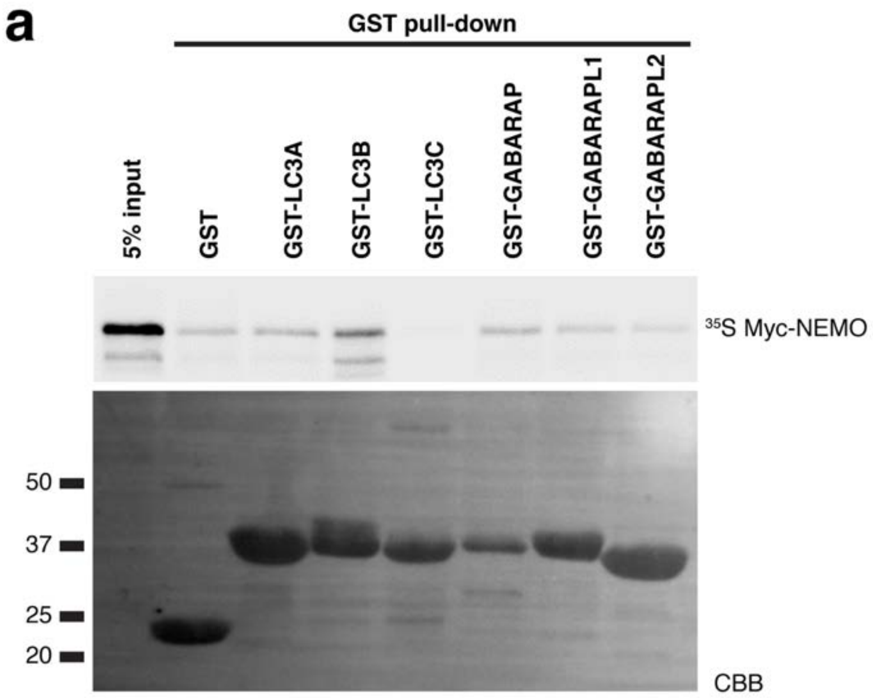

b Query: >sp|Q9Y6K9|NEMO_HUMAN NF-kappa-B essential modulator OS=Homo sapiens GN=IKBKG PE=1 SV=2

Motif	Start	End	Pattern	PSSM Score	LIR in Anchor
WxxL	232	237	VAYHQL	2	Yes
WxxL	338	343	REYSKL	11	No

Supplementary Figure 15. Human NEMO does not interact with ATG8s.

(a) GST-pulldown assays between mammalian GST-Atg8s (LC3A, LC3B, LC3C, GABARAP, GABARAPL1 and GABARAPL2) and radiolabelled myc-NEMO produced by coupled *in vitro* transcription and translation reaction in the presence of [^{35}S]methionine. **(b)** Screenshot of the result window from the iLIR search tool from the iLIR database (https://ilir.warwick.ac.uk) for the protein sequence of human NEMO (UniProt *Q9Y6K9*).

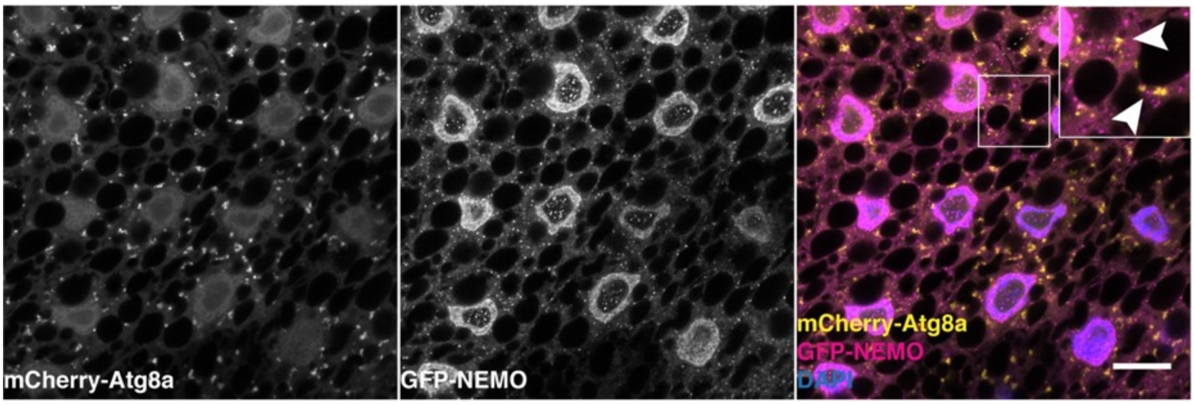

Supplementary Figure 16. Mammalian NEMO does not colocalize with Atg8a

Confocal sections of fat body from starved larvae expressing mCherry-Atg8a (yellow) and GFP-NEMO (magenta). DAPI was used to stain the nuclei. Arrowheads show that mCherry-Atg8a and GFP-NEMO puncta do not colocalize. Scale bar is 20µm.

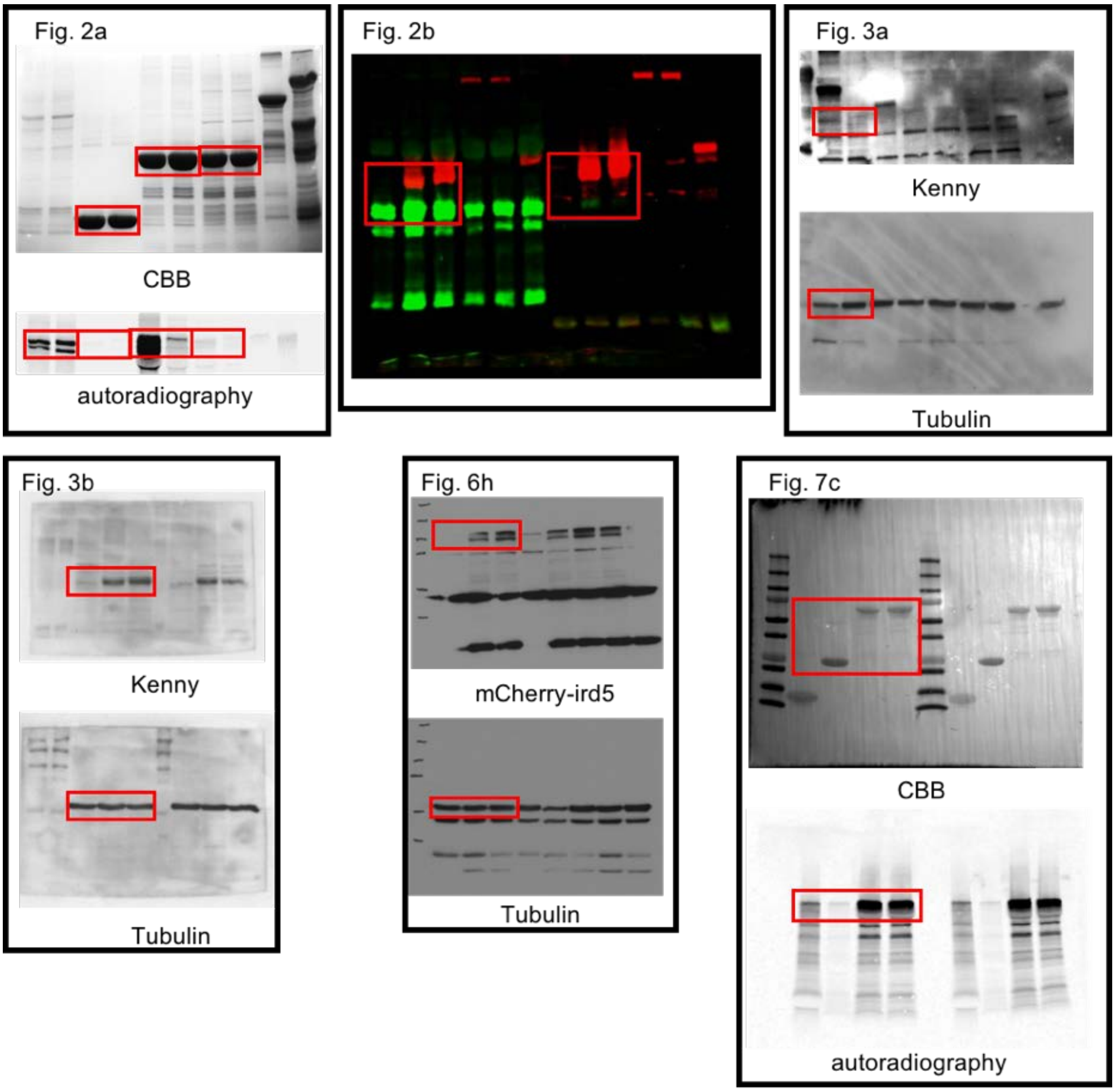

Supplementary Figure 17. Uncropped scans of western blots.

Cropped areas displayed in the main figures are framed in red.

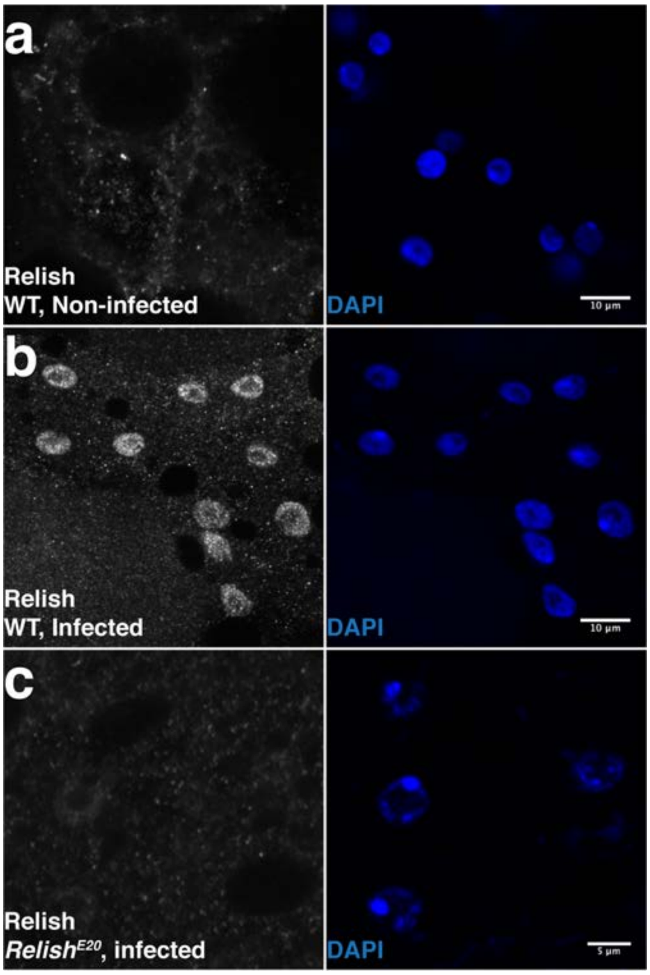

Supplementary Figure 18. Validation of Relish antibody

Confocal section of adult fat bodies stained for Relish (grey, left panels) and Hoechst (blue, right panels). (a) Wild-type flies non-infected. (b) Wild-type (WT) flies infected with *Ecc15*. (c) Rel^{E20} flies infected with *Ecc15*. Scale bars are 10µm (a, b) and 5µm (c).

Genotypes: (a, b) w^{1118}, (c) Rel^{E20}.

Supplementary Table 1. Genotypes of the flies used in the main figures Fig. 2 to Fig. 8

**Fig. 2**	
**c**	*yw hs-FLP/+;UAS-mCherry-Atg8a/+;Ac>CD2>GAL4/UAS-GFP-Kenny*^{WT}
**d**	*yw hs-FLP/+;UAS-mCherry-Atg8a/ UAS-GFP-Kenny*$^{F7A/L10A}$*;Ac>CD2>GAL4/+*
**Fig. 3**	
**a**	*yw Dpt-LacZ Drs-GFP*<br>*yw Dpt-LacZ Drs-GFP;key*^{1}
**b-l**	*w*^{1118}<br>*Atg8a*<br>*Atg7*$^{\Delta 14}$*/Atg7*$^{\Delta 77}$
**Fig. 4**	
**a**	*yw hs-FLP/+;tub-GAL4 UAS-GFP/+;FRT80B tub-GAL80/FRT80B Atg1*$^{\Delta 3D}$
**b**	*yw hs-FLP/+; FRT82 Ubi-GFP/FRT82 Atg13*$^{\Delta 81}$
**c**	*yw hs-FLP Ac>CD2>GAL4 UAS-mCD8-GFP/+;UAS-Atg5-RNAi/+*
**d**	*yw hs-FLP Ac>CD2>GAL4 UAS-mCD8-GFP/+;UAS-Atg8a-RNAi/+*
**Fig. 6**	
**a-d, h**	*Cg-GAL4 UAS-mCherry-ird5/+*
**e-g**	*Cg-GAL4 UAS-mCherry-GFP-ird5/+*
**Fig. 7**	
**a, d**	*Cg-GAL4 UAS-mCherry-ird5/+; UAS-GFP-Kenny*^{WT}*/+*
**b, e**	*Cg-GAL4 UAS-mCherry-ird5/UAS-GFP-Kenny*$^{F7A/L10A}$
**f**	*Cg-GAL4 UAS-mCherry-ird5/+; UAS-key RNAiv7723/+*
**g**	*Cg-GAL4 UAS-mCherry-ird5/+; UAS-luciferase RNAi/+*
**Fig. 8**	
	WT: *w*^{1118}<br>Atg7: *Atg7*$^{\Delta 14}$*/Atg7*$^{\Delta 77}$<br>Atg8a/+, *Atg8a/FM7c*<br>Atg8a/+;key^{1}: *Atg8a/FM6;key*^{1}*/key*^{1}.

Supplementary Table 2. Real-Time qPCR primers

	Forward primer sequence	Reverse primer sequence
GPDH	CCACTGCCGAGGAGGTCAACTA	GCTCAGGGTGATTGCGTATGCA
Diptericin	AGTTCACCATTGCCGTCGCC	GTAGGTGTAGGTGCTTCCCA
Kenny	GGGTTCATACCATCAGGCTAAA	CTGGCCTTCAGCTCGTTAAT
Ird5	TGACTCTCTACGCACGATAAAC	AATTGGATAAGCGGGCAATAAC
Atg8a	GGTCAGTTCTACTTCCTCATTCG	GATGTTCCTGGTACAGGGAGC
Atg7	TCGTGGGCTGGGAGCTAAATA	GGTTTACAGAGTTCTCAGCGAG

Supplementary Table 3. List of plasmids used

Plasmids	Source
pENTR-DmIKK/Kenny WT	This study
pENTR-DmIKK/Kenny F7A/L10A	This study
pDestMyc- DmIKK/Kenny WT	This study
pDestMyc-DmIKK/Kenny F7A/L10A	This study
pAFW-DmIKK/Kenny WT	This study
pAFW-DmIKK/Kenny F7A/L10A	This study
pPGW-Kenny WT	This study
pPGW-Kenny F7A/L10A	This study
pDest-mCherry-eYFP-Kenny WT	This study
pDest-mCherry-eYFP-Kenny F7A/L10A	This study
pDest15	Invitrogen
pDest15-Kenny	This study
pENTR-DmAtg8a	[1]
pENTR-DmAtg8a K48A/Y49A	[2]
pDest15-DmAtg8a	[1]
pDest15-DmAtg8a K48A/Y49A	[2]
pDestMyc-DmAtg8a	This study
pAGW-DmAtg8a	[2]
pENTR-DmIrd5	This study
pDestMyc-DmIrd5	This study
pPGW-DmIrd5	This study
pP(mCherry)W-DmIrd5	This study
pP(mCherry)GW-DmIrd5	This study
pDEST15-LC3A	[3]
pDEST15-LC3B	[3]
pDEST15-LC3C	[2]
pDEST15-GABARAP	[3]
pDEST15-GABARAPL1	[3]
pDEST15-GABARAPL2	[3]

Supplementary Table 4. Statistical description for the figures Fig.2 to Fig. 8

**Fig. 2**	
**e**	***$P<0.001$<br>two-tailed Student’s *t*-test
**Fig. 3**	
**c**	**$P<0.01$, ***$P<0.001$<br>one-way ANOVA test
**Fig. 7**	
**h**	**$P<0.01$<br>two-tailed Student’s *t*-test
**Fig. 8**	
**a, e, l, m**	*$P<0.05$, **$P<0.01$, ***$P<0.001$, ****$P<0.0001$<br>one-way ANOVA

Supplementary Table 5. This table details the values of parameters $\sigma_{x,i}$ and ψ_{xi} which were applied in the model in order to generate different combinations of effects, for the results shown in figure 9.

	No effects	Effect I only	Effect II only	Effect IIIa only	Effect IIIb only	Effect I and Effect IIIa	Effect II and Effect IIIa	Effect I and Effect IIIb	Effect II and Effect IIIb
ψ_{P1}	0.015	0.015	0.015	0.015	0.015	0.015	0.015	0.015	0.015
ψ_{P2}	0.015	0.015	0.015	0.015	0.015	0.015	0.015	0.015	0.015
ψ_{P3}	0.015	0.01	0.01	0.015	0.015	0.01	0.01	0.01	0.01
ψ_{P4}	0.015	0.01	0.011	0.015	0.015	0.01	0.011	0.01	0.011
ψ_{Q1}	0.015	0.015	0.015	0.015	0.01	0.015	0.015	0.01	0.01
ψ_{Q2}	0.015	0.015	0.015	0.015	0.015	0.015	0.015	0.015	0.015
ψ_{Q3}	0.015	0.01	0.01	0.015	0.01	0.01	0.01	0.005	0.005
ψ_{Q4}	0.015	0.01	0.011	0.015	0.015	0.01	0.011	0.01	0.011
σ_{P1}	0.7	0.7	0.7	0.7	0.7	0.7	0.7	0.7	0.7
σ_{P2}	0.7	0.7	0.7	0.7	0.7	0.7	0.7	0.7	0.7
σ_{P3}	0.7	0.7	0.7	0.7	0.7	0.7	0.7	0.7	0.7
σ_{P4}	0.7	0.7	0.7	0.7	0.7	0.7	0.7	0.7	0.7
σ_{Q1}	0.7	0.7	0.7	0.6	0.7	0.6	0.6	0.7	0.7
σ_{Q2}	0.7	0.7	0.7	0.7	0.7	0.7	0.7	0.7	0.7
σ_{Q3}	0.7	0.7	0.7	0.6	0.7	0.6	0.6	0.7	0.7
σ_{Q4}	0.7	0.7	0.7	0.7	0.7	0.7	0.7	0.7	0.7

SUPPLEMENTARY REFERENCES

1 Alemu, E. A. *et al.* ATG8 family proteins act as scaffolds for assembly of the ULK complex: sequence requirements for LC3-interacting region (LIR) motifs. *The Journal of biological chemistry* **287**, 39275-39290, doi:10.1074/jbc.M112.378109 (2012).

2 Jain, A. *et al.* p62/Sequestosome-1, Autophagy-related Gene 8, and Autophagy in Drosophila Are Regulated by Nuclear Factor Erythroid 2-related Factor 2 (NRF2), Independent of Transcription Factor TFEB. *The Journal of biological chemistry* **290**, 14945-14962, doi:10.1074/jbc.M115.656116 (2015).

3 Pankiv, S. *et al.* p62/SQSTM1 binds directly to Atg8/LC3 to facilitate degradation of ubiquitinated protein aggregates by autophagy. *The Journal of biological chemistry* **282**, 24131-24145, doi:10.1074/jbc.M702824200 (2007).
